# Dreaming Characteristics in Non-Rapid Eye Movement Parasomnia and Idiopathic Rapid Eye Movement Sleep Behaviour Disorder: Similarities and Differences

**DOI:** 10.2147/NSS.S435201

**Published:** 2024-03-08

**Authors:** Qi Rui See, Kausar Raheel, Iain Duncan, Nazanin Biabani, Irene Di Giulio, Andrea Romigi, Veena Kumari, David O’Regan, Scott Cairney, Daniele Urso, K Ray Chaudhuri, Valentina Gnoni, Panagis Drakatos, Ivana Rosenzweig

**Affiliations:** 1 Sleep and Brain Plasticity Centre, Department of Neuroimaging, Institute of Psychiatry, Psychology and Neuroscience (IoPPN), King’s College London, London, U.K; 2 School of Basic and Medical Biosciences, Faculty of Life Science and Medicine, King’s College London, London, U.K; 3 IRCCS Neuromed Istituto Neurologico Mediterraneo Pozzilli (IS), Pozzilli, Italy; 4 Centre for Cognitive Neuroscience, College of Health, Medicine and Life Sciences, Brunel University London, Uxbridge, U.K; 5 Sleep Disorders Centre, Guy’s and St Thomas’ NHS Foundation Trust, London, U.K; 6 Department of Psychology, University of York and York Biomedical Research Institute, University of York, York, U.K; 7 Center for Neurodegenerative Diseases and the Aging Brain, Department of Clinical Research in Neurology, University of Bari ‘Aldo Moro’, “Pia Fondazione Cardinale G. Panico”, Tricase, Lecce, Italy; 8 Movement Disorders Unit, King’s College Hospital and Department of Clinical and Basic Neurosciences, Institute of Psychiatry, Psychology & Neuroscience and Parkinson Foundation Centre of Excellence, King’s College London, London, U.K

**Keywords:** dreams, speech graph analysis, NREM parasomnia, REM behavior sleep disorder, dream content analysis

## Abstract

**Background:**

Speech graph analysis (SGA) of dreams has recently shown promise as an objective and language-invariant diagnostic tool that can aid neuropsychiatric diagnosis. Whilst the notion that dreaming mentations reflect distinct physiologic processes is not new, such studies in patients with sleep disorders remain exceptionally scarce. Here, using SGA and other dream content analyses, we set to investigate structural and thematic differences in morning dream recalls of patients diagnosed with Non-Rapid Eye Movement Parasomnia (NREMP) and Idiopathic REM Sleep Behavior Disorder (iRBD).

**Methods:**

A retrospective cross-sectional study of morning dream recalls of iRBD and NREMP patients was undertaken. Traditional dream content analyses, such as Orlinsky and Hall and Van de Castle analyses, were initially conducted. Subsequently, SGA was performed in order to objectively quantify structural speech differences between the dream recalls of the two patient groups.

**Results:**

Comparable rate of morning recall of dreams in the sleep laboratory was recorded; 25% of iRBD and 18.35% of NREMP patients. Aggression in dreams was recorded by 28.57% iRBD versus 20.00% in NREMP group. iRBD patients were more likely to recall dreams (iRBD vs NREMP; P = 0.007), but they also had more white dreams, ie having a feeling of having dreamt, but with no memory of it. Visual and quantitative graph speech analyses of iRBD dreams suggested stable sequential structure, reflecting the linearity of the chronological narrative. Conversely, NREMP dream reports displayed more recursive, less stable systems, with significantly higher scores of graph connectivity measures.

**Conclusion:**

The findings of our exploratory study suggest that iRBD and NREMP patients may not only differ on what is recalled in their dreams but also, perhaps more strikingly, on how dreams are recalled. It is hoped that future SGA-led dream investigations of larger groups of patients will help discern distinct mechanistic underpinnings and any associated clinical implications.

## Introduction

Dreams have been argued to present a state of consciousness that arises during sleep from an internally generated cognitive, sensory and emotional experiences.^1^ Commonalities between dreams and other stimulus-independent perceptions, such as hallucinations and mental imagery, comprise the internal mono- or poly sensory representation of an image within one’s mind.^2–4^ Unsurprisingly, some authors have argued that these perceptual phenomena may lie on a continuum, and that they may also utilize similar brain areas and pathways.^2^,^3^,^5^ Historically, dream reports linked with awakening from rapid eye movement (REM) sleep have been described as containing most complex, vibrant and intense experiences, whilst existence of dreams arising from non-REM (NREM) sleep has been negated or considered controversial.^1^ This has recently been disputed, and it has been shown that dreams arising from NREM sleep, albeit less frequently, may feature complex narratives and structured dreams.^5^,^6^

Moreover, recent evidence suggests that limited dreaming arising from NREM sleep may occur because of interfering phasic bursts or REM-like sleep microstructure.^7–11^ These phasic events have been linked to series of metabolically demanding physiologic events including contractions of the middle ear muscles, rapid eye movements, myoclonic twitches of skeletal muscles, sawtooth waves, irregularities in cardiorespiratory activity, and ponto-geniculo-occipital waves.^12^ Thus, phasic REM sleep likely plays a distinct role in dreaming. However, its function remains unclear with some linking it to the reactivation of vivid visuospatial mental pictures of specific emotional valence or to the reprocessing of emotional memories during sleep.^12^,^13^ Moreover, a body of work suggests that during sleep, an active systems’ consolidation process, embedded in global synaptic downscaling, may present the mechanistic platform for the formation of long-term memory.^14^ For instance, it has been proposed that during NREM slow-wave sleep, recurrent hippocampal neuronal-replays lead to the gradual transformation and integration of representations in neocortical networks.^14^

In this context, parasomnias have historically attracted the interest of sleep experts due to distinct sleep behaviors associated with dream mentation, and specific perceptual and sensory experiences.^15^,^16^ More specifically, sleep parasomnias, such as NREM parasomnia (NREMP)^5^,^9^,^17^,^18^ and (idiopathic) REM sleep Behavior Disorder (iRBD),^19^,^20^ exemplify a rare opportunity to gain a direct insight into dream activity and mentation.^1^,^6^,^16^,^21^

Parasomnias are associated with distinct sleep states/stages, but they can also occur during sleep wake transitions.^6^ RBD, characterized by loss of typical REM-like atonia and abnormal behaviors during REM sleep, may indicate an early stage of neurodegeneration linked with alpha-synucleinopathies.^22^ Most of abnormal motor events likely arise during phasic REM, which in RBD can vacillate from elementary movements to vivid dream enactments.^8^,^23^ Historically, RBD was predominantly linked with male sex,^24–27^ but that has been increasingly disputed with recognition that women tend to have a significantly later age of onset of RBD^28^,^29^ and that they less frequently report dream-enacting behaviours.^29–31^ Fernández-Arcos et al reported significantly more aggressive behavior in male RBD, and less so in women, who dreamt more about children in life-threatening contexts.^30^ Thus, prevalence of female RBD might be grossly underestimated in the community. Here, the lack of specific questionnaires that would in equal measure also account for differential female RBD behaviours may also play a part.^32^ Conversely, NREM parasomnias, marked by significant physical action and autonomic activation, appear to be common at all ages, with slightly higher incidence in children and younger adults.^1^ Patients can injure themselves during acted out NREM events, and they can endanger themselves and others.^11–13^ Excessive daytime sleepiness is frequently reported by NREM parasomnia sufferers, as well as lower threshold for pain and poorer quality of life.^9^,^13^

Understanding how basic physiological components of sleep may contribute to the genesis of dreams will be pivotal for future translational clinical purposes.^1^,^7^,^21^,^32^ However, it is widely accepted that oneiric research is significantly limited by an inaccessible nature of dreams, where any insight on intrinsic dream activity depends on retrospective recall by dreamers.^6^,^33^ These, as of yet non-circumvented, methodological problems effectively mean that oneiric research commonly incorporates data that has unknown quantity of distortions in recall, along with omissions caused by reprocessing of memory for the event.^1^,^6^,^34^,^35^ Thus, perhaps unsurprisingly, dream research remains in its infancy, and to date only one another study has formally investigated putative differences between dream contents that arise from NREM parasomnia or RBD events.^6^ This study found that dreams of RBD patients were more complex and had a higher incidence of aggression. In particular, authors reported that NREMP events may predominantly translate into a “flight” defensive response to any perceived threat, whereas RBD patients were shown to engage in violence and counterattack more commonly when assaulted. In this background, we set to investigate the dream contents of patients with idiopathic RBD (iRBD) and NREMP, all of whom had a verified parasomnic event via video polysomnography (PSG). To define any putative distinct cognitive biomarkers, patients’ recalls of dreams upon awakening that they believed to be associated with the parasomnic event were collected, analyzed and compared according to their thematic contents, complexity, and content differences. Moreover, in order to objectively explore iRBD and NREMP dream report structure, a speech graph analysis (SGA) was subsequently undertaken.^36^

## Materials and Methods

An exploratory study was conducted between May 2018 and January 2020, during which period all clinically diagnosed iRBD and NREM parasomnia patients who underwent video-polysomnography (VPSG) at the Sleep Disorders Center (Guy’s Hospital, London, United Kingdom) were identified.

### Research Design

**Figure 1:**
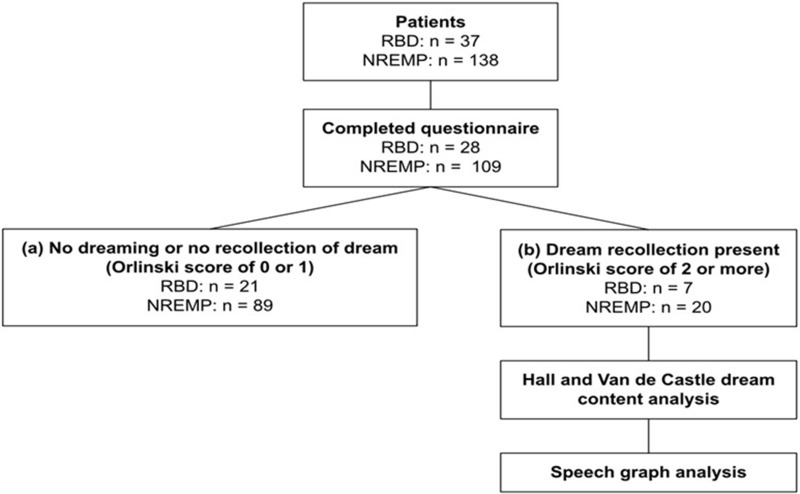
Flowchart of the studied cohort of patients with iRBD and NREM parasomnia.

**Abbreviations**: n, total number of patients; NREMP, non-REM (NREM) parasomnia; iRBD, idiopathic rapid eye movement (REM) sleep behaviour disorder.

Patients diagnosed with iRBD (n = 37) and NREM parasomnia (n = 138) according to the American Academy of Sleep Medicine (AASM) criteria, as previously published^5^,^8^ (also please refer to Figure 1), were initially identified. Of those initially identified, only seven iRBD and 20 NREMP patients had fulfilled required inclusion criteria for speech graph analysis (Orlinsky score of 2 or above), and thus subsequently their dream reports and sleep macrostructures were analyzed; none were receiving any psychotropic medication at the time of the VPSG investigation. The study was approved by the Guy’s and St Thomas’ Hospital Clinic Research Ethics Committee (Project-No-9585 and Project-No-11,378, GSTT NHS, UK), which did not require informed patients’ consent for retrospectively ascertained anonymized data where the study protocol was judged to abide by the strictest patients’ data confidentiality and when it complied with the EU General Data Protection Regulation and with the Declaration of Helsinki.

### Clinical Review and Morning Dream Questionnaire

Systematic review of patients’ medical records, VPSG recordings, and post-VPSG morning questionnaires was undertaken, as previously described.^5^ As a part of an established clinical routine at the Sleep Disorders Centre, patients were asked to fill the post-VPSG morning questionnaires upon awaking, in order to record any oneiric mentation linked with their diagnostic VPSG-recorded motor events. The morning questionnaires were semi-structured, self-administered, and contained open-ended questions with elements of both the Orlinsky scale^37^ and the Hall and Van de Castle dream coding system.^38^ For instance, participants were asked whether they were able to remember dreams related to their parasomnic event, the emotions and people associated with them, and how they felt after dreaming.

### Methods of Analysis

#### Orlinsky Score Analysis

Based on the participants’ responses to the morning dream questionnaire, their mental content was scored using the Orlinsky scale^37^,^39^ (further details are provided in Supplementary Table 1). The dreams were rated from 0 to 7 (0 = no dream recall; 1 = feeling of having dreamt but no memory of it; 2 = specific topic in isolation; 3 = disconnected thoughts, scenes, or actions; 4 = short but coherent dream; 5 = detailed dream sequence with two stages; 6 = long, detailed dream sequence with three or four stages; and 7 = detailed dream sequence of five or more).^39^ For instance, for this analysis, if a patient reported not having remembered dreaming, it was scored as 0.^39^ Positive responses to questions related to dreaming were scored as 1. Conversely, if a patient reported dreaming and was able to describe the dream, it was scored as 2 or above depending on its content.^39^ A detailed explanation of the Orlinsky scoring system is provided in the Supplementary Table 1. The terms coherent/incoherent here suggest faulty articulation of a consistent dream narrative, either in relation to the dream mentation, chronology of events, or both.

According to the patients’ Orlinsky scores, they were further divided into two subgroups: (a) those with Orlinsky scores of 0 or 1 and (b) those with Orlinsky scores of 2 or above. Only those in subgroup (b) had dream reports, which were further evaluated using Hall and Van de Castle^38^ dream analysis and speech graph analysis.^36^

#### Hall and Van de Castle Dream Content Analysis

Dream reports of patients who scored 2 or above 2 on the Orlinsky scale (ie, seven iRBD and 20 NREMP patients) were subsequently analyzed using the Hall and Van de Castle method.^4^ The Hall and Van de Castle method is considered as one of the most comprehensive systems for studying dream content.^4^ It comprises ten general categories, most of which are further divided into two or more subcategories. In this study, based on an initial pilot analysis of the patients’ dream content, nominal categories, and more specifically, categories for characters, emotions, and social interactions, including aggression, were used to qualify the dream contents. No dream records consisted of content that would be classified under the categories of friendliness, sexuality, fortune and misfortunes; thus, these categories were excluded from further analysis.

#### Speech Graph Attributes (SGA) Analysis

In order to investigate the structural organization of the iRBD and NREMP dream reports, we used speech graph attributes (SGA) Analysis.^36^ Specifically, 14 graph-theoretical attributes were derived and evaluated in the context of the total report length (word count).

SGA analysis has already shown significant promise as a non-invasive, affordable, and language-invariant tool for psychiatric diagnosis.^36^ SGA analysis enables identification of most promising behavioral biomarkers that can subsequently guide a bottom-up search for more objective neuroanatomical and neurophysiological biomarkers (also refer to).^36^

**Figure 2:**
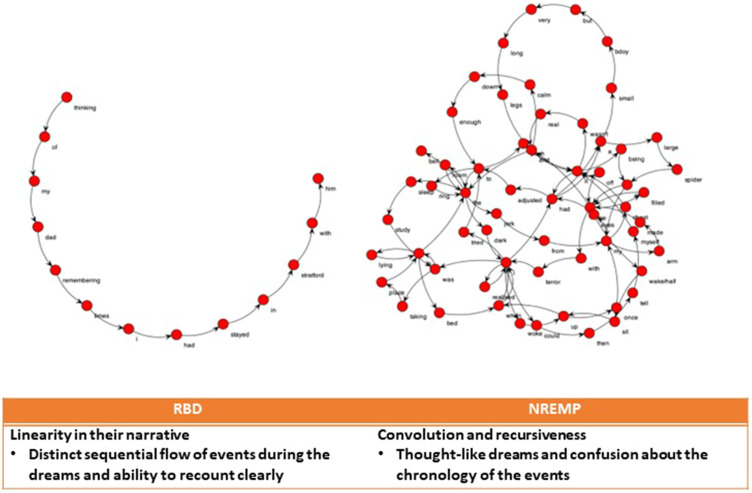
Representative speech graph transcribed from the dream reports of one iRBD and one NREMP patient. Adapted from Mota NB, Vasconcelos NA, Lemos N, et al. Speech graphs provide a quantitative measure of thought disorder in psychosis. *PLoS One*. 2012;7(4):e34928. Creative Commons.^36^

**Abbreviations**: NREMP, non-rapid eye movement parasomnia; iRBD, idiopathic rapid eye movement sleep disorder.

Speechgraphs, free Java software (http://neuro.ufrn.br/softwares/speechgraphs), were used to transcribe the dream reports into non-semantic word graphs for our speech graph attribute analysis. To investigate the structural organization of iRBD and NREMP dream reports, each dream report was represented as a word graph, where nodes represent the words used and edges correspond to semantic and grammatical relationships.^36^ A number of graph measures were derived, as previously published. We concentrated on measures of recurrence and global, general and connectivity graph attributes (see Figures 2 and 3) as complementary tools to specifically investigate the non-semantic structural organization of RBD and NREMP dream reports.^36^ Here, for example, in cases of two hypothetical separate reports, one with high recursiveness and higher interconnectivity, compared to the other, more linear report, with minimal recursiveness in a speech structure, one would expect higher scores for the difference between two connectivity parameters, namely the largest strongly connected component (LSC) and the largest connected component (LCC). In a purely linear graph, these attributes are the same, but marked differences between them arise from interconnectivity and recursiveness.

In previous work, a number of graph theoretical measures, such as graph connectedness and graph random likeness, were found to be useful predictors of organizational changes in psychiatric disorders such as schizophrenia.^36^,^40^

**Figure 3:**
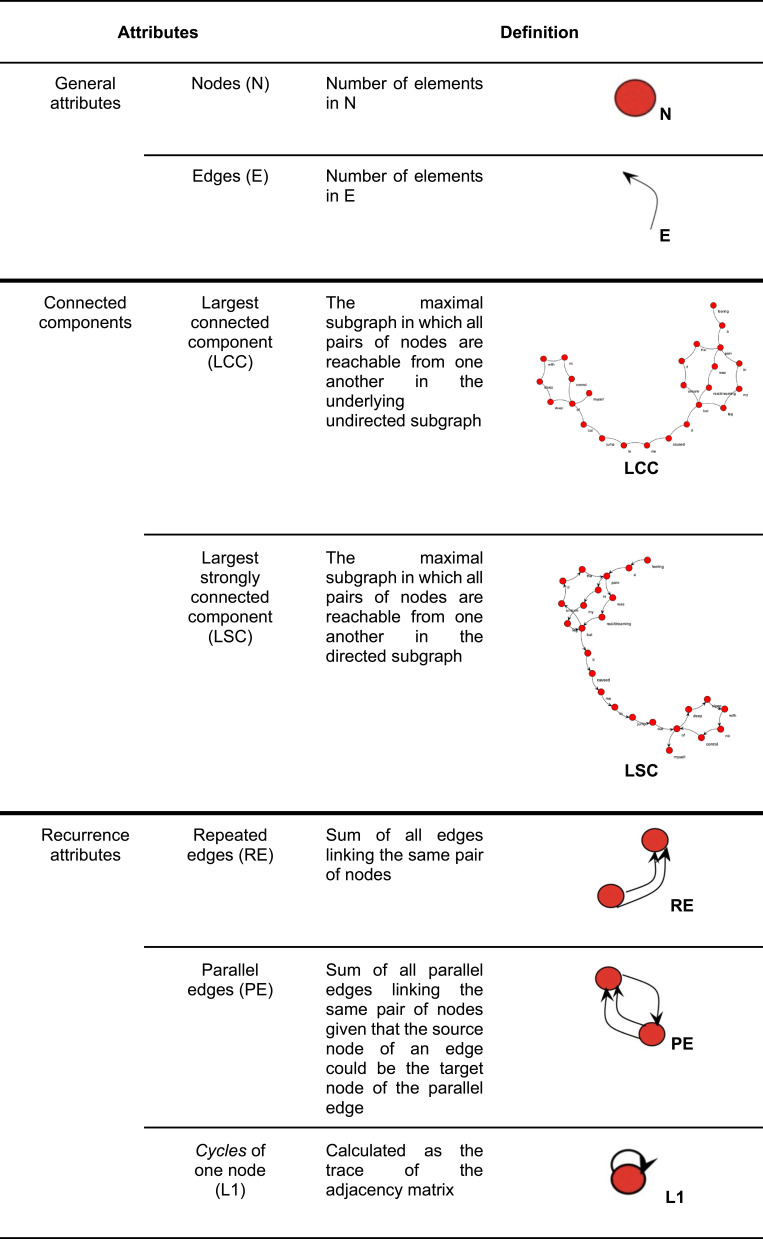
Continued.

**Figure 3:**
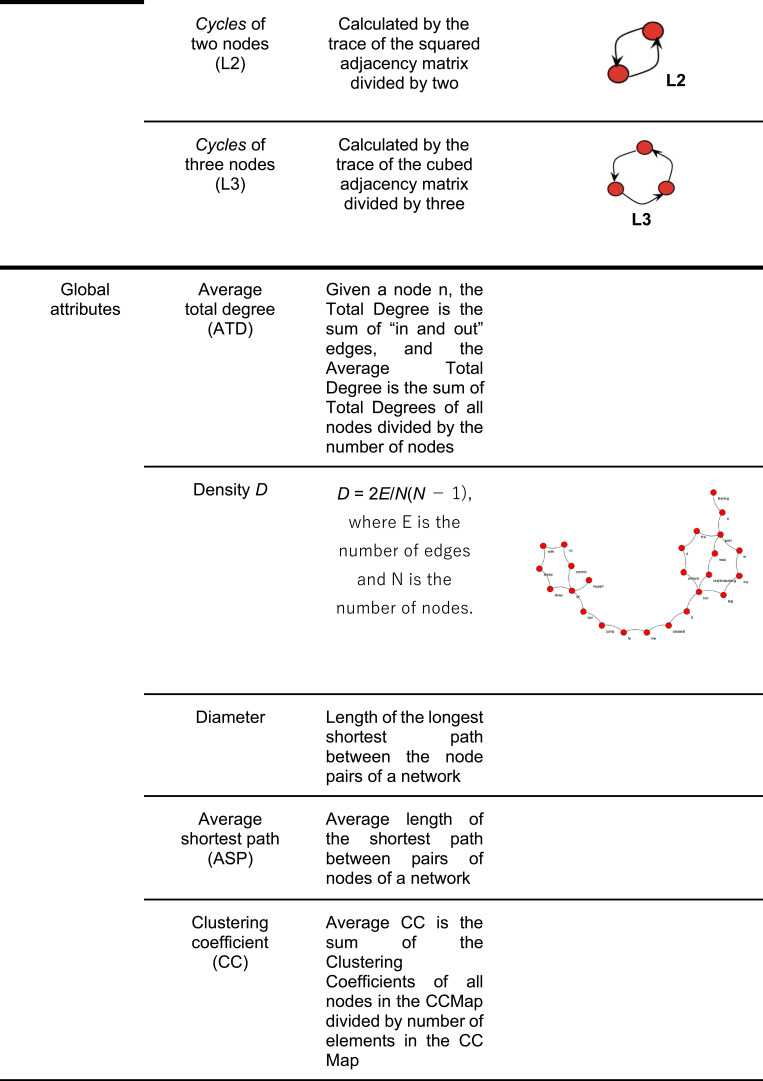
Speech graph attributes (SGA) and their definitions adapted from Mota NB, Vasconcelos NA, Lemos N, et al. Speech graphs provide a quantitative measure of thought disorder in psychosis. *PLoS One*. 2012;7(4):e34928. Creative Commons.^36^

### Statistical Analysis

IBM SPSS Statistics (version 28.0.0.0) was used to analyze the data. All categorical data were reported as frequencies and percentages. Owing to the small sample size, Fisher’s exact test was used to compute the *P* value for the differences between the two groups of patients. The test was two-tailed, and a *P* value of 0.05, was considered significant. For polysomnographic variables and demographic group comparisons, we used one-way ANOVAs for parametric variables, Mann–Whitney *U*-tests for non-parametric variables, and Pearson’s χ^2^ tests for categorical variables.

For graph speech analysis, 14 speech graph attributes were normalised by the number of nodes, tested for Gaussian distribution (Kolmogorov–Smirnov test of normality). Speech graphs were subsequently visually inspected, following which comparisons of graph speech attributes between the two groups were performed using either parametric (one-way ANOVA) or non-parametric statistics (Mann–Whitney *U*-test).

## Results

The rate of morning recall of dreams in the sleep laboratory was similar in the iRBD and NREMP groups (7/28 in iRBD *versus* 20/109 in NREMP, *P*=0.432).

### Participants

Sociodemographic and polysomnographic descriptive statistics of all iRBD and NREMP patients who scored above two on the Orlinsky scale are shown in Table 1.

**Table 1:** Socio-Demographic and Sleep Parameters for iRBD and NREM Parasomnia Patients with Dream Recollection

Total Number of Patients	iRBD		NREMP		Statistics	
	7		20			
	Mean	SD	Mean	SD	F	*p*
Age (years)	32.0	7.37	35.3	9.32	0.714	0.406
BMI (kg/m^2^)	29.6**	4.04**	25.2	4.00	5.561	0.027*
	**n**	**%**	**n**	**%**	**χ^2^**	***p***
Gender (female)	4	57.1	11	55.0	0.306	0.580
	**Median**	**IQR**	**Median**	**IQR**	**U**	***p***
**Sleep Macrostructure**						
TST (min)	360	35.5	378	68.5	55.0	0.407
WASO (min)	52.7	89.0	62.3	69.3	67.0	0.868
SL (min)	16.5	17.0	17.5	16.1	65.0	0.782
REM L (min)	113	145	104	116.75	67.5	0.890
NREM1 (%)	7.3	4.5	12.1	5.85	35.5	0.056
NREM2 (%)	47.1	16.1	47.1	9.85	44.0	0.580
NREM3 (%)	25.1	19.7	19.7	7.55	27.0	0.017*
REM%	18.3	8.3	21.6	9.85	68.0	0.912
AI	13.4	4.9	17.7	7.7	34.0	0.046*
AHI	3.0	7.6	0.95	3.0	57.5	0.488
PLMI	9.0	10.2	2.45	5.45	38.5	0.077
	**n**	**%**	**n**	**%**	**χ^2^**	***p***
**Other Sleep Disorders**						
RLS	1	14.30	3	15.00	0.002	0.963
Hypnopompic Hallucination	1	14.30	1	5.00	0.652	0.419
Bruxism	0	0.00	3	15.00	1.181	0.545
Sleep paralysis	0	0.00	3	15.00	1.181	0.545
OSA	0	0.00	2	10.00	0.756	1.000
Insomnia	0	0.00	2	10.00	0.756	1.000
PLMD	0	0.00	1	5.00	0.363	1.000
Hypnagogic Hallucination	0	0.00	1	5.00	0.363	1.000

**Notes**: *Denotes statistically significant differences (P < 0.05). **Denotes that one of the iRBD patients was excluded in this calculation because their BMI was not accessible during the study. Group comparisons were performed using one-way ANOVAs for parametric variables, Mann–Whitney U-tests for non-parametric variables, and Pearson’s χ^2^ tests for categorical variables.

**Abbreviations**: AHI, apnea–hypopnea index; AI, arousal index; BMI, body mass index kg/m^2^; F, one-way ANOVA; IQR, interquartile range; n, number; NREM, non-rapid eye movement; OSA, Obstructive Sleep Apnea; p, p-value; PLMD, Periodic Leg Movement Disorder; REM, rapid eye movement; REM, rapid eye movement latency; RLS, Restless Leg Syndrome; SL, sleep latency; SD, standard deviation; TST, total sleep time; U, Mann–Whitney U statistics; WASO, wake after sleep onset.

Dream reports of seven iRBD patients (42.9% male) and 20 NREMP patients (45% male) were further evaluated. The participants were age-matched and their ages ranged between 20 and 59 years, with a mean age of 32.0 (SD) = 7.37) and 35.3 (SD = 9.32) years for the iRBD and NREMP groups, respectively (*P* = 0.406). The BMI of iRBD patients was significantly higher than that of NREMP patients (29.6 ± 4.04 versus 25.2 ± 4.00 kg/m^2^, *P* = 0.027). However, sleep- and non-sleep-related comorbidities did not differ between sleep and non-sleep related comorbidities (Table 1).

### Orlinsky Score

Twenty-one iRBD and 89 NREM patients did not report any dreams and thus scored below 2 on the Orlinsky score (see Table 2 for further details). Patients with iRBD were more likely to report having a feeling of having a dreamt, but with no memory of it (white dream), in comparison to patients with NREMP (*P* = 0.007).

**Table 2:** Orlinsky Scores of iRBD and NREMP Patients

	iRBD		NREMP		*p*
	*f*	*%*	*f*	*%*	
Total no. of patients	21		89		
0. No dream recall	5	23.81	52	58.43	0.007*
1. Feeling of having dreamt but no memory of it	16	76.19	37	41.57	0.007*
2. Specific topic in isolation or fragments	4	57.14	14	70.00	0.652
3. Several disconnected thoughts, scenes or actions	0	0.00	2	10.00	1.000
4. Short but coherent dream	2	28.57	2	10.00	0.269
5. Detailed dream sequence, 2 events or stages occur	1	14.29	2	10.00	1.000
6. Long detailed dream sequences, 3 or 4 distinct stages	0	0.00	0	0.00	1.000
7. Extremely long sequence of 5 or more stages	0	0.00	0	0.00	1.000

**Notes**: *(in bold) denotes statistically significant differences (p < 0.05). iRBD, idiopathic rapid eye movement sleep disorder; group comparisons were conducted using Fisher’s exact test.

**Abbreviations**: %, percentage; *f*, frequency; NREMP, non-rapid eye movement parasomnia; p, p-value.

### Sleep Macrostructure

Several differences were observed in the gross sleep macrostructure between the two patient groups. For instance, while duration of sleep was similar in the two patient groups (TST: 360 ± 35.5 versus 378 ± 68.5 minutes respectively, *P* = 0.407), the percentage of time spent in stage N3 was recorded significantly longer for iRBD patients (n = 7; 25.1 ± 19.7 versus n = 20 NREMP patients, 19.7 ± 7.55%, respectively, *P* = 0.017). Conversely, the arousal index was significantly higher in NREMP patients (17.7 ± 7.7 versus 13.4 ± 4.9, respectively, *P* = 0.046).

### Hall & Van de Castle Dream Content Analysis

#### Characters

The proportions of animals and humans reported by both groups were similar. NREMP patients (66.67%) frequently reported dreaming of indefinite characters (Table 3), eg, those whose sex or gender was unidentifiable from the dream content. Common terms used in dream reports include “someone”, “a person”, and “a tall figure”.

**Table 3:** Characters Frequency and Percentage According to the Hall & Van de Castle Dream Content Analysis

	iRBD		NREMP		*p*
	*f*	*%*	*f*	*%*	
**Characters**	7		19		
Animals	0	0.00	2	10.52	1.000
Creatures	1	14.29	2	10.52	1.000
Human	6	85.71	15	78.95	1.000
Group human	0	0.00	5	33.3	0.262
Single human	6	100.00	10	66.67	0.262
Indefinite	1	16.67	10	66.67	0.063
Male	3	60.00	1	20.00	0.523
Female	2	40.00	4	80.00	0.523
Familiar	2	33.33	1	6.67	0.184
Unfamiliar	4	66.67	14	93.33	0.184
Family	2	33.33	1	6.67	0.184

**Notes**: Explanation of terms used in this Table: characters, animals, creatures, and humans refer to the total number of characters, animals, creatures, and humans dreamt, respectively; Group human refers to the total number of characters that consist of two or more individuals who are not individually identified; Single human refers to the total number of characters that are described as individuals; Indefinite refers to the total number of characters whose genders are unidentifiable; Male and Female are the total number of male and female characters, respectively, and percentages are relative to the total number of definite human characters; Familiar refers to the total number of characters that are in one of the following four identity subclasses: family, relatives, Known, and Prominent; Unfamiliar refers to the total number of characters that are in the following identity subclasses: Occupational, Ethnic, Uncertain, and Stranger; Family is the total number of characters coded as family members or relatives. The male/female percentage was calculated by dividing the number of male characters by the total number of male and female characters; Familiarity percentage was calculated by dividing the number of familiar characters by the total number of familiar and unfamiliar characters; family percentage was taken to be the total number of family members or relatives out of total humans; Group percentage was the total number of grouped characters out of total humans; Animal percentage was taken to be the total number of animal characters out of total characters.

**Abbreviations**: %, percentage; *f*, frequency; NREMP, non-rapid eye movement parasomnia; iRBD, rapid eye movement sleep disorder.

iRBD patients (60.00%) more often reported dreaming of male characters than NREMP patients (20.00%), but this did not reach statistical significance. They also reported dreaming of familiar characters or their family members more often than did patients with NREMP. Animals were similarly frequently reported in both groups.

#### Social Interaction, Aggression and Emotions

The percentage of dreams in which aggression occurred was similar for both groups (28.57% in iRBD versus 20.00% in NREMP group).

Regarding reported emotions, only negative emotions were recorded. Specifically, apprehension was reported by both groups and was the only emotion that was discernible in the iRBD group. In NREMP patients, the prevalent emotion was apprehension (66.67%), whilst confusion and sadness were also reported.

Further breakdown of patients’ responses and major indicators are found in Tables 2–4.

**Table 4:** Hall & Van de Castle Analysis of Social Interaction, in Particular, Aggression

	RBD		NREMP	
	f	%	f	%
Dreams in which aggression occurs	2	28.57	4	20.00
Total instances of aggression	4		5	
Dreamer-involved aggression	3	75.00	5	100.00
Fight response	2	66.67	1	20.00
Flight response	1	33.33	4	80.00
**Major indicators**				
A/C Index	0.44		0.26	
Aggressor (%)	0.00		0.00	
Physical Aggression Percent (%)	25.00		60.00	

**Notes**: *Explanation of terms used*: Dreamer-involved aggression refers to the number of instances in which the dreamer was involved in aggression; fight response refers to the number of instances in which the dreamer retaliated or engaged in mutual aggression; flight response refers to the number of instances in which the dreamer receives aggression without retaliation and/or escapes from the situation; and A/C index is the Aggression-per-Character index, calculated by dividing the total instances of aggression by the total number of characters. The aggregate percentage was calculated by dividing the number of instances in which the dreamer initiated an aggressive act by the total number of instances of aggression. The percentage of physical aggression was the number of instances in which aggression was physical, out of the total instances of aggression.

**Abbreviations**: %, percentage; *f*, frequency; NREMP, non-rapid eye movement parasomnia; iRBD, rapid eye movement sleep disorder.

### Speech Graph Analysis (SGA)

The dream reports with scores above 2 on the Orlinsky scale (seven iRBD and 20 NREMP) were further analysed using speech graph analysis, as previously described.^36^ Visual inspection of speech graphs suggested distinct structural differences between the two groups (Figure 2). For instance, iRBD dream reports, with a mean word count (SD) of 23.43 (17.71) words, were typically sequential with little recursiveness, suggesting the linearity of the chronological narrative. Conversely, dream reports of patients with NREMP parasomnia, with a mean word count of 28.15 (16.35) words, displayed patterns of convolution, higher interconnectivity, system instability and recursiveness, predictive of higher scores for the largest strongly connected component (LSC), a graph connectivity and recursiveness measure in SGA.

Accordingly, the findings of nonparametric Mann–Whitney U analyses of normalised (per number of nodes) LSC demonstrated significant difference between the iRBD and NREMP dream reports [Mann Whitney U = 109, P = 0.033; effect size r = 0.779, z = 2.16, η2 = 0.172]. Normalised LSC was significantly higher in NREMP group (M = 0.57, SE = 0.063), compared to iRBD group (M = 0.295, SE = 0.069). Post hoc analysis of other attributes did not reveal any other statistically significant trends; please refer to Supplementary Tables 2–4 for a detailed report.

## Discussion

The principal findings of our small exploratory study suggest similar rates of morning dream recall between (young) middle-aged iRBD and NREMP patients who have experienced a PSG-verified nocturnal parasomnic event. Moreover, we reported a higher percentage of white dreams^41^ in iRBD patients and a higher rate of NREMP patients with no recall of dreams.

Historically, white dreams have been described in terms of problems with encoding or retrieval^42^ and defined by the feeling of having had an experience of dreaming whilst unable to account for that experience.^41^ More recently, however, it has been suggested that white dreaming may simply signify lower-quality dream experiences where neural activity is higher than in no dream recall condition but lower than in cases of dream recall.^41^,^43^ Thus, some white dreaming might result from “contentless” or “imageless” dreams, a minimal form of consciousness, with subjects experiencing no narrative structure.^41^ Arguably, it would follow that in iRBD, even in the early stages of neurodegeneration, distinct changes in sleep microstructure and a neurochemical/neurocircuitry milieu may contribute to less vivid dreams’ mental imagery, and therefore, a higher rate of recall of white dreams by some individuals.^8^,^19^,^20^ Similarly, a higher rate of no dream recall in NREMP might be caused by ensuing down-states during NREM slow-wave sleep and even less well-defined dreams’ mental imagery.^44^

In keeping with this notion, characters recalled by NREMP patients were less well defined (Hall & Van de Castle Dream Content Analysis: indefinite characters: 66.7% versus 16.7% in iRBD), and their overall dream recall significantly less coherent (eg, SGA analysis). Previously, we and others have suggested that the subconscious processing of external environmental cues during NREMP episodes may promote discontinuity and ambiguity in the mental imagery of dreamers.^5^,^9^,^10^,^43^ In this NREMP cohort, however, dream recall may have been further affected by significant sleep fragmentation, as evidenced by a higher arousal index compared to iRBD patients. Moreover, as shown in Figure 2, the NREMP reports in our study demonstrate high convolution and recursiveness, thought-like dreams, and confusion regarding the chronology of events. This is in striking opposition to the temporally less ambiguous dream narrative of the iRBD group (eg, with story-like linearity and easily recounted distinct sequential flows of events).

Notably, the SGA analysis indicated that NREMP dreamers had to repeatedly revert to internally experienced snapshot mental imagery while building a dream narrative (Supplementary Figure 1). Alternatively, these findings could be attributed to a specific pattern of memory deficits with amplified emotional salience (for example, arising during associated pavor nocturnus or panic attacks).^10^ All of this could theoretically affect NREMP patients’ recall, more than a similar request put to iRBD patients.

Overall, we propose that our results support the feasibility of automatic differential diagnosis of dream narratives based on word-by-word speech graph analysis (Figure 2).^36^ Interestingly, it has been shown that schizophrenia and bipolar affective disorder patients, both previously associated with significant changes in sleep architecture,^21^,^45^ respond in a reciprocal manner to the dream-report task. For instance, the former were reported to sustain flattened speech, whereas the latter responded to increased confabulation.^36^ A body of work on other neuropsychiatric syndromes also suggests that dream-reports may be significantly more revealing than waking reports about the mental state of patients.^36^ Thus, the use of SGA may further aid true holistic treatment approaches.^1^,^36^,^44^

In addition, the findings of our study further converge on previously reported fundamental principles of diverse processing across distinct sleep brain states.^1^ For instance, while the recent waking activities (episodic replay) may be reported in only 1–2% of dream reports, it has been shown that retrieval of declarative memories may be more accessible after REM sleep.^36^ Thus, depending on the neurochemical milieu and changes in sleep architecture that underlie the majority of sleep or neuropsychiatric disorders, dream reports may become more or less likely to be proximally anchored to recent events, while simultaneously also more or less likely to reveal psychopathologies based on mature memories, likely to be restructured over time.^36^,^44^

To the best of our knowledge, this is only the second study to date that directly compared the dream characteristics of these two parasomnias.^6^ Moreover, while our findings are in broad agreement with previous findings,^1^,^46^ there are also notable differences that likely reflect the distinct sociodemographic characteristics of our younger (and age-matched) iRBD and NREMP patient cohorts. For example, we reported a similar rate of dream recall between the two parasomnias, which is in contrast to a previous study that reported a higher recall of dreams in NREMP patients (vs iRBD).^6^ However, in an earlier study, parasomnia cohorts were not age-matched, and (much younger) NREMP patients were compared with a significantly older RBD group.^6^ Interestingly, the authors did not find any differences when dream recall was assessed over a lifetime.^6^ This may further support the role of age and possibly the neurodegenerative effect on short-term memory capacity, and thus on recall.^46–48^ Moreover, whilst both patient cohorts were diagnosed according to the strict American Academy of Sleep Medicine (AASM) criteria, as previously published,^5^,^8^ it is important to note that our iRBD cohort included uncommonly young patients, of which more than half were women (Table 1). This unusual phenotype likely reflects that dream recall may be age and sex dependent, and thus, these data may not be fully representative of older, male iRBD patients. Reassuringly, and in broad keeping with our results, one well-conducted video-polysomnographic study of iRBD, showed that dream content was linked to video-recorded motor behaviours above chance level.^35^

Previous work has suggested that oppositional defensive isomorphic flight or fight defence behaviors may arise in NREMP and iRBD patients under a perceived threat during dreaming, and similar behavioral patterns have been observed in this study.^6^ However, the small sample size and other inherent limitations of our observational retrospective study, which relied on self-administered questionnaires,^38^,^39^ prevented further in-depth analysis of this phenomenon (Table 4).^1^ In the past, proactive counterattack fighting defence mechanisms recorded in iRBD dreams have been compared to more avoidant (flight response) defence reactions in sleep terrors (NREMP).^1^ The former is mechanistically linked to the disinhibition of archaic defence behaviors stemming from central pattern generators.^49^,^50^ Interestingly, a recent study has suggested a negative link between age and levels of aggression in dream mentation in healthy participants, which has also been confirmed in patients with phenoconverted RBD.^51^ Therefore, it is possible that indirect consequences of any such episode (eg, injuries, spousal/partner impact) in RBD patients play a significant role in enhancing the long-term memory of the incident.^51^

Another interesting finding in our study is that both groups of patients had proportionally similar numbers of reported dream characteristics (7/7 for iRBD and 19/20 for NREMP). However, characters recalled by iRBD patients appeared better defined, which may be a reflection of the vividness of the mental imagery of dreams during REM sleep that allows for better identification of the participating characters.^1^ However, it is also possible that this is due to inherent differences in dream imagery arising across tonic and phasic stages of the NREM-REM spectrum.^7^

Moreover, examining the extremes of nightmares, such as in cases of post-traumatic stress disorder (PTSD), sufferers commonly report experiencing a torturous replay of their trauma through nightmares and can often identify their attackers as their dreamt character.^52^ The ventral visual stream, a neurocircuitry pivotal for object recognition and categorization, has been shown to play a role in the development, severity, and response to nightmares in patients with PTSD and is likely to play an active role in the character identification process in nightmares.^53^ In one of our previous studies on iRBD patients, we highlighted the restricted truncal sagittal movements of enacted dreams with eyes closed as a reflection of allocentric navigation (brain-generated virtual space-maps), which involves maps that are represented/recorded in the hippocampal area, which are functionally linked with the ventral visual stream and amygdala.^54^ Allegorically, iRBD patients could be likened to the viewer of a 3D cinema movie, who watches a film directed by allocentric dream landscapes, requiring no external spatio-temporal coordinates or input, and thus possibly more easily retrieving familiar characters from existing memory engrams. The seated viewer may react to a 3D movie only restrictively.^8^ In contrast, and in parallel, in NREMP dream mentations, egocentric spatial maps are utilized, with eyes typically open during parasomnia events, enriching their dream content and navigation process with visual information from the surroundings, all of which may generate mental imagery/perceptions of unspecified characters and gender, originating, for instance, from a shadow behind the bedroom door or at the ceiling.^6^,^55^

Although our sample size prevents any authoritative exploration of putative sex differences in dream mentations, we note that aggression in male iRBD patients was predominantly reported as the same sex directed. For example, in this study, male patients were more likely to counterattack other male characters. Conversely, male NREMP patients were more likely to flee or avoid a female character during a perceived threat. Previous studies on sex differences in aggressive dream content between healthy college males and females have suggested heightened aggression in dreams of male dreamers, with perceived dream attackers reported more frequently as males.^56^

In conclusion, dream research, including this study, has many overt significant limitations inherent to poorly defined neuroscientific definitions of sleep stages, dreams and mental imagery, lack of reproducible findings, and sufficiently developed imaging methodologies. Finally, the role of memory in accessing experiences with no objective means by which dreams can be collected and independently validated poses additional unresolved issue.^1^,^41^,^57^ Just as dream reports of our two parasomnia cohorts cannot be authoritatively linked to any specific sleep stage, there is no objective way to determine whether the morning recall by NREMP patients is that of NREM or possibly of REM dream origin or possibly a confabulation, and vice versa.^57^ Moreover, any extrapolation of our findings is further limited by its small size, as well as its observational nature.

Notwithstanding, the findings of SGA quantitatively characterized RBD and NREMP cohort differences, and future well-defined studies with much larger samples will hopefully build on some of these hypotheses. Such studies will need to use longitudinal measures on the same participants, ideally while using various reproducible dream study protocols, combined with multimodal imaging.^1^
